# Hippocampal TNF-death receptors, caspase cell death cascades, and IL-8 in alcohol use disorder

**DOI:** 10.1038/s41380-020-0698-4

**Published:** 2020-03-05

**Authors:** Wen Liu, Ryan P. Vetreno, Fulton T. Crews

**Affiliations:** Bowles Center for Alcohol Studies, University of North Carolina at Chapel Hill, Chapel Hill, NC 27599-7178 USA

**Keywords:** Neuroscience, Molecular biology

## Abstract

The relationship between increased neuroimmune gene expression and hippocampal degeneration in alcohol use disorder (AUD) and other mental diseases is poorly understood. We report here that tumor necrosis factor receptor superfamily death receptor 3 (TNFRSF25, DR3) and Fas receptors (Fas) that initiate caspase cell death cascades are increased in AUD hippocampus and following a rat adolescent binge drinking model. Death receptors are known inducers of apoptosis and cell death that recruit death domain (DD) proteins FADD and TRADD and caspases to form death-inducing signaling complexes (DISC). In postmortem human AUD hippocampus, mRNA and IHC protein are increased for the entire death receptor cascade. In AUD hippocampus, ligand–death receptor pairs, i.e., TL1A-DR3 and FasL–Fas, were increased, as well as FADD and TRADD, and active caspase-8, -7, -9, and caspase-3. Further, *p*NFκB p65, a key neuroimmune transcription factor, and IL-8, a chemokine, were significantly increased. Interestingly, across AUD patients, increases in DR3 and Fas correlated with TRADD, and TRADD with active caspase+IR and IL-8+IR, consistent with coordinated activation of neuronal DISC mediated death cascades and neuroimmune gene induction in AUD. These findings support a role for DR3 and Fas neuroimmune signaling in AUD hippocampal neurodegeneration.

## Introduction

Neurodegeneration and increased expression of neuroimmune genes are common to many brain diseases including Alzheimer’s disease and Parkinson’s disease [1], depression [2], and alcohol use disorder (AUD) [3]. Neuroimmune genes are signaling proteins discovered as innate immune signals that are expressed in brain. How these signals cause neurodegeneration is poorly understood. Humans with AUD suffer from global subtle neurodegeneration and reduced hippocampal volumes associated with deficits in cognition and increased negative affect [4, 5]. Preclinical studies in rats find alcohol exposure decrease hippocampal dentate gyrus (DG) neurogenesis [6] and increase markers of DG-induced neuronal death including: classic H&E, TUNEL, silver stain, Fluoro Jade B staining, and electron microscopy-identified necrotic morphology as well as immunohistochemistry (IHC) for active (cleaved) caspase-3+IR (CC3+IR) [7–10]. Alcohol exposure also increases brain neuroimmune gene expression, which mimics that found in postmortem human AUD brain and is hypothesized to contribute to alcohol-induced neurodegeneration [3, 11] and the development of AUD [12–14]. Postmortem human AUD brain has increased expression of proinflammatory cytokines, chemokines, and Toll-like receptors (TLRs) as well as HMGB1, an agonist at these immune receptors, and oxidases and other immune signaling factors in the prefrontal cortex, with many correlating with lifetime alcohol consumption [15–17]. However, as in other neurodegenerative diseases, the mechanisms of alcohol-induced neuroimmune signaling and neurodegeneration in the hippocampus are unclear. Recent studies have employed CC3+IR, i.e., the cleaved activated protease form of the enzyme, as an “executioner caspase” carrying out apoptotic, necroptotic and/or necrotic cell death [18, 19]. Models of adolescent binge drinking find increased neuroimmune gene expression and markers of cell death in DG, including CC3+IR expression, that appear to cause a persistent reduction in adult hippocampal neurogenesis and MRI-determined volume [7–9, 20–22] as well as increased adult anxiety, negative affect, and alcohol drinking [23]. Interestingly, a unique subset of neuroimmune receptors is known to be linked to caspase death cascades, i.e., the death receptors (DRs), suggesting DR signaling may contribute to hippocampal degeneration.

DRs are a subset of the tumor necrosis factor receptor superfamily (TNFRSF) that contain a unique protein sequence known as the death domain (DD). DRs initiate a death-inducing signaling complex (DISC) of proteins that share DDs leading to CC3-induced cell death through multiple paths. The DD-containing receptors death receptor 3 (DR3, TNFRSF25) and Fas receptor (Fas, FasR, CD95, TNFRSF6) are expressed at low levels in the hippocampus of rodents and humans [24]. The TNFSF agonists TNF-like 1A (TL1A, TNFSF15, VEGI) for DR3 and Fas ligand (FasL, TNFSF6, CD95L) for FasR, are also expressed in brain, as are components of the DISC, i.e., the DD-signaling protein Fas-associated death domain (FADD) and caspase-8, an initiator caspase. Tumor necrosis factor receptor type 1-associated death domain protein (TRADD) is also linked to ligation-induced complexes of DR–TRADD–FADD that activate caspase cascades of cell death. These DISC complexes regulate cell death by recruiting the initiator protease caspase-8, which can cleave itself with DD association, initiating cleavage of executioner caspases. Executioner caspase-3 and caspase-7 undergo cleavage-induced protease activation, leading to multiple forms of regulated cell death [18, 19]. DR3 and Fas also signal through kinases that activate nuclear factor (NF)-κB transcription and induction of immune and other genes that are associated with cell death and neurodegenerative diseases. Most studies of these signaling pathways have been done on immune cells, although all of these proteins are expressed at low levels in adult brain on both glia and neurons, where they have been suggested to contribute to brain development [24], autoimmune encephalitis [25], amyotrophic lateral sclerosis [26], and stroke pathology [27]. Transgenic mice lacking DR3 and Fas show normal gross brain development to adulthood followed by age-related systemic immune changes and unusual brain regional degeneration [24, 28]. Our previous findings of adolescent alcohol exposure increasing adult hippocampal activated CC3+IR led to the hypothesis that alcohol-induced persistent DR signaling contributes to AUD hippocampal pathology.

We report here that the expression of DR signaling proteins is increased in postmortem AUD hippocampus and adolescent ethanol-exposed rat hippocampus. Markers of DR signaling correlate across AUD patients consistent with DR-caspase cascades contributing to AUD neuroimmune gene induction and hippocampal alcoholic neurodegeneration.

## Materials and methods

### Animal experiment procedures

Wistar rats were ordered from Harlan Laboratories Inc. (Indianapolis, IN, USA) under a protocol approved by the Institutional Animal Care and Use Committee at UNC Chapel Hill as described previously [9]. On P21, male Wistar rats (*n* = 8/group) were randomly assigned to either: (i) AIE or (ii) water control (CON) conditions. To minimize the impact of litter variables, no more than one subject from a given litter was assigned to a single experimental condition. From P25 to P54, AIE subjects received a single daily intragastric (i.g.) administration of ethanol (5.0 g/kg, 20% ethanol, w/v) in the AM on a 2-day on/2-day off schedule, and CON subjects received comparable volumes of water on an identical schedule. Following treatment animals matured without additional exposure and were sacrificed on P95. During AIE exposure, tail blood samples were collected 1 h after ethanol exposure at P38 and P54 with average blood ethanol values of 152 ± 11 mg/dl at P38 and 212 ± 19 mg/dl at P54. At the conclusion of experimentation, subjects were anesthetized and tissue collected as previously described (see [15]).

### Postmortem AUD human hippocampus specimens

Postmortem human hippocampus (*n* = 10/group) was provided by the New South Wales Tissue Resource Centre at the University of Sydney in Australia which provided both paraffin sections and frozen tissues. The detailed patients’ medical history and subject information is presented in Supplementary Table 1. Previous studies have detailed NSWTRC phenotyping and tissue collection details [29, 30].

### Immunohistochemistry (IHC) staining and quantification

For all antigens, antibodies were validated (see Supplementary Table 2) and assessed on human paraffin sections. Sections were deparaffinized in xylene and rehydrated in a series of ethanol, and antigen retrieval was completed. Free-floating rat brain and rehydrated human hippocampal tissue samples were incubated in 0.6% H2O2 for 30 min and blocked in 5% goat serum (0.1% Triton X-100; Sigma-Aldrich, St. Louis, MO, USA) for 1 h at room temperature (RT). Sections were incubated with primary antibodies overnight at 4 °C. Primary antibodies, dilutions and validation information are included in Supplementary Table 2. After overnight incubation, sections were washed and incubated with biotinylated secondary goat anti-rabbit or goat anti-mouse antibody (1:200, Vector Laboratories, Burlingame, CA, USA) at RT for 1 h. Subsequently, avidin–biotin–peroxidase complex (ABC Elite Kit, Vector Laboratories) was applied for 1 h at RT, and then the positive cells were visualized using 3,3′-diaminobenzidine (DAB; Sigma-Aldrich). BioQuant Nova Advanced Image Analysis (R&M Biometric, Nashville, TN, USA) was used for image capture and analysis using a modified stereology method previously described [31]. Slides were coded for blind quantification. For rats, 3–5 sections of the dorsal DG (bregma from −2.12 to −4.52 mm) were quantified as described previously [9].

### RNA isolation and quantitative reverse transcription polymerase chain reaction (RT-qPCR)

Total mRNA was extracted from frozen hippocampal tissue sections from human AUD and moderate drinking controls by homogenization in TRI reagent (Sigma-Aldrich) following the single step method [32]. Total mRNA was reverse transcribed as previously described [15, 17]. SYBER green PCR Master Mix (Life Technologies) was used for the RT-qPCR. The real-time PCR was run with an initial activation for 10 min at 95 °C, followed by 40 cycles of denaturation (95 °C, 15 s), annealing/extension (57–58 °C, 1 min), and finally a melt curve. The primer sequences are presented in Supplementary Data Table 3. The threshold cycle (CT) of each target product was determined and the ΔΔCT method was used to calculate the percent change relative to moderate drinking controls. All samples were run in triplicate. The CT of each target product was determined and normalized to internal standard β-actin. RT-qPCR CT values of the housekeeping gene β-actin did not differ between control (23.4 ± 0.2) and AUD (23.8 ± 0.2) subjects (*p* > 0.10).

### Statistical analysis

Statistical analysis was performed using SPSS (Chicago, IL). Sample size determinations were based on previously published studies [9, 14, 15]. No subjects were excluded from the analyses. Independent samples *t*-tests were used to assess IHC and RT-qPCR data between control and AIE or humans with AUD and their matched control group. Levene’s test for equality of variances was performed for each analysis. Pearson correlations were used to determine the correlation across all markers in the hippocampal DG. Data are reported as mean ± SEM. The difference was considered significant at *p* < 0.05. Pearson correlations were used to determine the correlation across all markers in the hippocampal DG.

## Results

### Ethanol exposure induces persistent increase of DRs and ligands in adult hippocampal DG

**Fig. 1:**
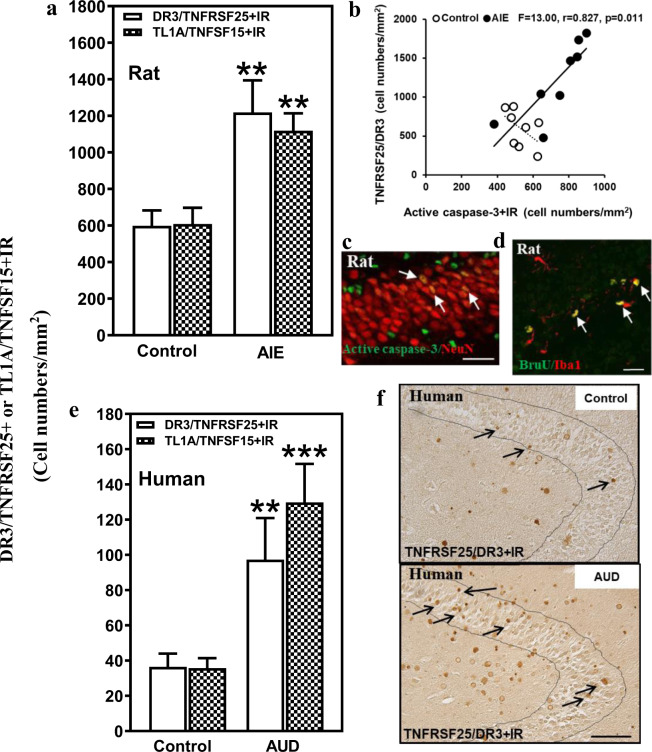
Hippocampal expression of DR3 and the DR3 ligand TL1A in a rodent model of adolescent intermittent ethanol (AIE) and postmortem human alcoholic hippocampus. **a** Effects of AIE (5.0 g/kg, i.g. 2 days on, 2 days off, postnatal day [P]25–54) on DR3/TNFRSF25+IR or TL1A/TNFSF25+IR expression in the male rat hippocampal dentate gyrus (DG) at P95. AIE exposure increased DR3/TNFRSF25+IR expression (204% of control) compared with control group. AIE exposure also increased TL1A/TNFSF15+IR expression (184% of control). The data were expressed as the numbers of DR3/TNFRSF25+ or TL1A/TNFSF15+ positive cells. Each point is mean ± SEM per mm^2^ (*n* = 8/group). **b** The correlation between DR3+IR and CC3+IR expression in control and AIE groups in the DG of rat brain. **c** Photomicrographs of confocal images in the hippocampal DG, active caspase-3+ (green) and NeuN+ (red), bar scale = 30 μm. **d** Increased DR3/TNFRSF25+IR and TL1A/TNFSF15+IR expression in the DG of postmortem AUD human brain. DR3/TNFRSF25+IR (267% of control) and TL1A/TNFSF15+IR (364% of control) expression in the AUD DG were significantly increased compared with control. Data were expressed as the numbers of DR3/TNFRSF25+ or TL1A/TNFSF15+IR positive cells. Each point is mean ± SEM per mm^2^ (*n* = 10/group). **p < 0.01 compared with control group by independent samples *t*-test. **e** DR3+IR expression in the DG of postmortem human hippocampus (immunohistochemical staining, bar scale = 50 μm).

To determine if ethanol exposure alters expression of DR3/TNFRSF25, we studied a rat model of underage binge drinking previously found to persistently increase neuronal death in hippocampal DG in association with increased expression of multiple neuroimmune genes as well as active CC3 and other markers of adult hippocampal neuronal death [8, 9]. We found that AIE exposure more than doubled DR3+IR expression in the rat DG (204%, *p* < 0.01, Fig. 1a) and increased expression of the DR3 ligand TL1A/TNFSF15+IR (184%, *p* < 0.01, Fig. 1a). TL1A-DR3 signals through DD-containing proteins, e.g., TRADD and FADD, to CC3 inducing cell death. We found AIE exposure also increased adult DG CC3+IR expression (138% of control, *p* < 0.01, not shown) as previously described [24]. Expression of DR3+IR cells correlated with expression of DG activated CC3+IR cells (Fig. 1b). In addition, CC3+IR colocalized with NeuN, a neuronal marker, consistent with induction of DR3 increasing neuronal CC3-mediated cell death. These findings suggest that long-lasting increases in DR3 could contribute to increased CC3+IR.

### Expression of DRs and ligands in human hippocampal DG

**Fig. 2:**
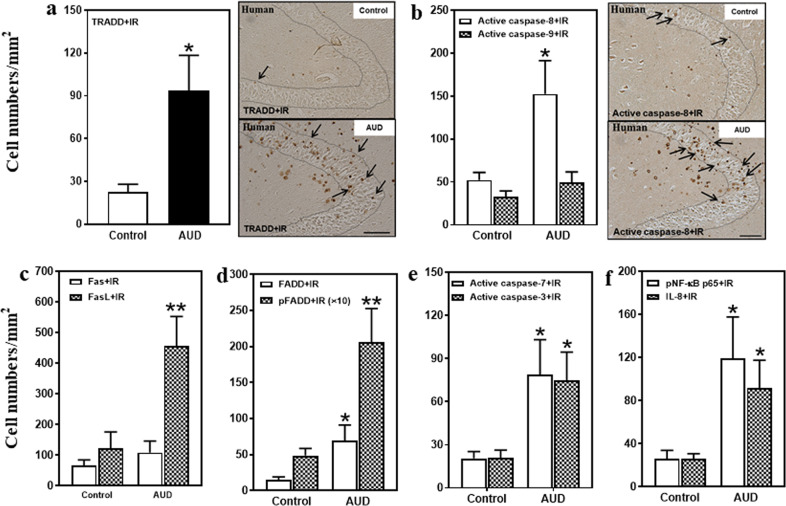
Increased protein expression of death receptor signaling in the hippocampal dentate gyrus (DG) of postmortem AUD human brain. **a** Left side: TRADD+IR expression was significantly increased (407% of control) in the AUD DG. Right panel: TRADD+IR expression in the DG of postmortem human hippocampus (immunohistochemical staining, bar scale = 50 μm). **b** Right side: Active caspase-8+IR expression was increased (292% of control), but there was no change in active caspase-9+IR expression in the AUD DG. Right panel: Active caspase-8+IR expression in the DG of postmortem human hippocampus (immunohistochemical staining, bar scale = 50 µm). **c** FasL+IR expression was significantly increased (370% of control) in the AUD DG. However, there was not significant change in Fas+IR expression. **d** FADD+IR (469% of control) and pFADD+IR (424% of control) expression were significantly increased in the AUD DG. **e** Active caspase-7+IR (385% of control) and active caspase-3+IR (356% of control) expression were significantly increased in the AUD DG. **f** pNF-κB p65+IR (342% of control) and IL-8+IR (347% of control) expression were significantly increased in the AUD DG. Data are expressed as the numbers of cells. Each point is mean ± SEM per mm^2^ (*n* = 9–10/group). *p < 0.05, **p < 0.01 compared with control group with independent samples *t*-test.

Although AUD is known to reduce hippocampal volume, the mechanisms of neurodegeneration are poorly understood. To determine the expression of TL1A-DR3 as well as FasL–Fas in human hippocampus and the impact of alcohol abuse, we compared ten moderate drinking controls to ten AUD individuals matched by age and multiple other characteristics (see Supplementary Table 1). Expression of TL1A and DR3 as well as FasL and Fas was low in control DG. Interestingly, the expression of DR3/TNFRSF25+IR in AUD DG was increased 267% (Fig. 1d, Supplementary Fig. 1a, *p* < 0.01), and TL1A/TNFSF15+IR expression was increased by 395% of control (Fig. 1d, *p* < 0.01; Supplementary Fig. 1b). Assessment of TL1A and DR3 mRNA similarly found increased expression in AUD to 248% and 324% of control, respectively (see Table 1). We also investigated FasL–Fas, another ligand–DR pair, and found FasL+IR expression increased 370% (*p* < 0.01, Fig. 2c, Supplementary Fig. 1c) and mRNA 285% in AUD (*p* < 0.01, Table 1). Although Fas mRNA was increased 258% (*p* < 0.05) in AUD (Table 1), Fas+IR expression, which assesses protein levels, was not statistically different in AUD (162%, Fig. 2c). Since we found ethanol exposure in rats caused a persistent increase in TL1A-DR3 signaling in rat hippocampus, our findings of increases in human AUD hippocampus are likely due to alcohol drinking.

**Table 1:** Expression of cell death signaling gene mRNA in the postmortem human AUD hippocampus.

*Gene*	Control	AUD	Independent samples *t*-test statement
*Fas*	100 (±22)	258 (±61)*	*t*(18) = 2.4, *p* < 0.05
*FasL*	100 (±23)	285 (±49)***	*t*(18) = 3.4, *p* < 0.01
*DR3*	100 (±18)	324 (±93)*	*t*(18) = 2.3, *p* < 0.05
*TL1A*	100 (±25)	248 (±74)	*t*(18) = 1.9, *p* = 0.07
*FADD*	100 (±9)	195 (±37)*	*t*(18) = 2.5, *p* < 0.05
*TRADD*	100 (±21)	266 (±87)	*t*(18) = 1.9, *p* = 0.08
*IL-8*	100 (±25)	243 (±68)	*t*(18) = 0.67, *p* = 0.52
*Caspase 3*	100 (±20)	251 (±68)*	*t*(18) = 2.1, *p* < 0.05
*Caspase 7*	100 (±19)	284 (±86)*	*t*(18) = 2.1, *p* = 0.05
*Caspase 8*	100 (±20)	269 (±62)*	*t*(18) = 2.6, *p* < 0.05
*Caspase 9*	100 (±24)	267 (±83)	*t*(18) = 1.9, *p* = 0.07
*NF-κB p65*	100 (±19)	256 (±72)*	*t*(18) = 2.1, *p* = 0.05

Data are presented as % of moderate drinking controls with mRNA (RT-PCR) values. Presented as mean (±S.E.M.). See Supplementary Table 2 for case characteristics of human subjects.

*p  ≤  0.05, ***p  <  0.005.

Death receptors like DR3, upon ligation by agonists, recruit DD adapter proteins (i.e., FADD and TRADD) that form DISC that activate caspase cascades. We found FADD+IR and phosphorylated FADD (pFADD)+IR were expressed at low levels in controls that in AUD hippocampus were increased 469% (*p* < 0.05) and 424% (*p* < 0.01), respectively (Fig. 2d, Supplementary Fig. 1d). Similarly, FADD mRNA in AUD hippocampus was increased 195% (*p* < 0.05; Table 1). TRADD+IR expression in AUD was increased 414% (*p* < 0.05; Fig. 2a), relative to control. Increases in FADD+IR, pFADD+IR, and TRADD+IR expressing cells in postmortem AUD hippocampus further support increased TL1A-DR3 and FasL–Fas signaling, and DISC–DD complex signaling.

DISC activate cell death caspase cascades [33, 34]. Caspases are proteases formed as inactive proenzyme zymogens that upon proteolytic cleavage become active proteases. To determine if caspases were activated in AUD, we used antibodies to the active cleaved caspase proteases. DISC initiator caspases (e.g., caspase-8 and caspase-9) undergo self-proteolytic activation as well as proteolytic activation of cell executioner caspases such as caspase-7 and caspase-3 [35]. AUD hippocampus was found to have twofold to threefold more caspase-8, -7 and -3 mRNA (Table 1). AUD hippocampal active caspase-8+IR cells were increased 292% (*p* < 0.05; Fig. 2b), active caspase-7+IR were increased 385% (*p* < 0.05; Fig. 2e), and active caspase-3+IR expression increased 356% (*p* < 0.05; Fig. 2e; Supplementary Fig. 1e). These findings are consistent with increased expression and activation of DISC cell death caspases in AUD hippocampus.

In our previous studies, we found AUD increased brain expression of proinflammatory cytokines, and that adolescent alcohol exposure of rats also increases proinflammatory gene expression in hippocampus [13]. We assessed phospho-NFκB p65+IR, a transcriptionally active form of this key proinflammatory transcription factor, and also extended studies to assess the chemokine interleukin-8 (IL-8), which has recently been implicated in alcohol craving [36]. We found low expression in controls, but in AUD DG, pNFκB p65+IR expression increased to 481% (*p* < 0.05) and IL-8+IR expression increased to 347% (*p* < 0.05) of control (Fig. 2f; Supplementary Fig. 1f, g). NFκB p65 mRNA was increased 256% in AUD (*p* < 0.05; Table 1). These findings are consistent with increased expression of proinflammatory genes in postmortem human hippocampus that could be triggered in part through DR3 and/or Fas signaling or might result from AUD-induced neurodegeneration [18, 19].

### Correlations of DR, TRADD, and caspase cascade in AUD hippocampal DG

**Fig. 3:**
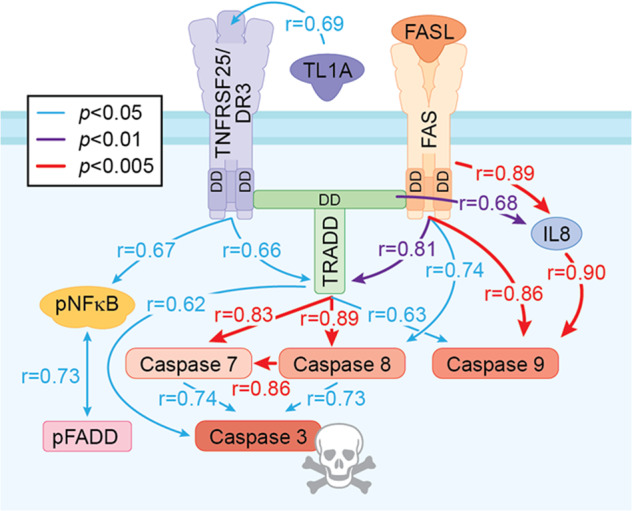
Correlation of multiple markers of cell death signaling pathway in the hippocampal dentate gyrus (DG) of postmortem AUD human brain. Correlation analysis with significant Pearson correlation of AUD-induced activation cell death signaling pathway across all protein markers used in the DG. Only statistically significant correlations are shown. Blue solid line indicates statistically significant correlation *p* value < 0.05; purple solid line indicates that *p* value < 0.01; red solid line indicates that *p* value < 0.005. The value of correlation coefficient *r* is located across the line between markers.

TNF ligand-receptor signaling is characterized by induction and amplification of ligands, receptors, and associated cascade signaling proteins within cells [34, 35]. Interestingly, across AUD patients DR markers had multiple significant correlations across endpoints whereas this was rare in controls (Table 2, Fig. 3). Figure 3 is a diagram of the DR3-TRADD-FADD caspase cascade with statistical correlation values colored for significance in AUD hippocampus. In particular, TRADD+IR showed significant correlations with expression of all four active caspase+IR assessed as well as DR3+IR (*p* < 0.05), Fas+IR (*p* < 0.01), and IL-8+IR (*p* < 0.05), with caspase-8+IR (*p* < 0.01) and caspase-7+IR (*p* < 0.01) being highly correlated with TRADD+IR. Although correlations do not establish causal relationships, the correlations across AUD patients are consistent with coordinated expression and activation of death receptor signaling. Similarly, in AUD Fas+IR was significantly correlated with TRADD+, caspase-8+, caspase-9+, and IL-8+IR, consistent with known signaling cascades. These findings are consistent with increased hippocampal Fas and DR3 DISC caspase signaling in postmortem AUD hippocampus.

**Table 2:** Correlations across immunohistochemical markers of cell death signaling in the postmortem human hippocampus of moderate drinking controls (C) and individuals with AUD (A).

	TNFSF15/TL1A	TRADD	Cleaved caspase 3	Cleaved caspase 7	NF-κB p65	Fas	IL-8
TNFRSF25/DR3	0.69* (A)	0.66* (A)	-	-	0.67* (A)	-	-
pFADD	-	-	-	-	0.73* (A)	0.62* (C)	-
Cleaved caspase 3	-	0.62* (A)		0.74* (A)	-	-	-
Cleaved caspase 7	-	0.83*** (A)	0.74* (A)		-	-	-
Cleaved caspase 8	-	0.89*** (A)	0.73* (A)	0.86*** (A)	-	0.74* (A)	-
Cleaved caspase 9	0.81** (C)	0.63* (A)	-	0.81** (C)	-	0.86*** (A)	0.90*** (A)
Fas	-	0.81** (A)	-	-	-		-
FasL	-	-	-	-	0.75* (C)	-	-
IL-8	-	0.68* (A)	-	-	-	0.89*** (A)	-

Pearson's *r* correlations were conducted to assess the association of cell death signaling genes in postmortem human hippocampal tissue samples from moderate drinking controls and individuals with AUD. Pearson’s r correlation coefficients were used with two-tailed significance and were assessed between groups. (A) indicates AUD and (C) indicates controls. Lack of correlation is designated by (-). There were no significant correlations of IHC across all patients, only within AUDs or controls as indicated.

*p  ≤  0.05, **p  <  0.01, ***p  <  0.005.

## Discussion

We report here that postmortem human hippocampus from individuals with AUD and rats exposed to ethanol have increased expression of DRs and activated caspase cascades known to induce apoptosis and other forms of cell death [18, 19]. To our knowledge, this is the first time human hippocampal histochemistry of DISC proteins, DR cascade components, and mRNAs has been linked to AUD hippocampal degeneration. Previous studies of postmortem human AUD hippocampus have found increased expression of proinflammatory cytokines, IL1B, and CCL2 (MCP1)[37, 38], as well as TLR7 and HMGB1 [39]. We report here for the first time an increased expression of IL-8, a cytokine linked to AUD and alcohol craving [40], adding new data on increased innate immune gene expression in AUD hippocampus. Human imaging studies have found adults with AUD [4] as well as adolescents with AUD [41] have reduced hippocampal volume, suggesting that heavy drinking contributes to hippocampal pathology. Studies in rodents have similarly found that adolescent alcohol exposure causes a persistent loss of adult neurogenesis with increases in cell death [7–9] that may contribute to a loss of adult hippocampal volume [8, 20]. We found AUD expression of the TL1A and DR3 as well as FasL gene and protein were increased several-fold. TL1A/TNFSF15 was initially identified as a factor inducing vascular endothelial cell apoptosis [42] and this finding has been extended to immune cells, cancers and, in this study, to hippocampal neurons. TL1A and DR3 generally are known for low basal expression with most research focused on lymphocytes and autoimmune diseases [42], although previous studies have identified TL1A mRNA in mouse brain [28]. A characteristic of TNF receptor signaling is amplification of ligands, receptors, and cascade signaling components within and across cells; in brain this likely involves glia and neurons [3, 24]. We found TRADD, FADD, and pFADD expression increased about fourfold, consistent with amplification of TL1A, DR3, and FasL initiating TL1A-DR3-TRADD-FADD-procaspase 8 complexes referred to as DISC ((DISC, 27)). This complex leads to cleavage and activation of caspase 8, the initiator caspase of death cascades [19]. Using antibodies to activate proteases, we found increased caspase-3, -7, and -8+IR protein and mRNA gene expression, supporting activated protease cell death cascades in AUD postmortem human hippocampus. These findings support DR3 and Fas-mediated cell death, a receptor-activated mechanism that differs from glutamate excitotoxicity, which is an additional known mechanism of degeneration [27]. Cerebral ischemia triggers induction of TNF-death receptor and Fas signaling in rat brain that peaks between 24 and 48 h, which coincides with the time course of apoptosis [27]. Transgenic mice expressing dysfunctional Fas or TNF as well as neutralizing antibodies reduce cerebral infarct size [43]. Our finding of increases in cells expressing TL1A-DR3 and FasL–Fas, signaling proteins FADD, TRADD, and NFκB as well as initiator and executioner caspases are consistent with alcohol exposure induction of an apoptotic cascade that correlates across endpoints in AUD, but not controls. These findings suggest apoptotic neuroimmune signaling contributes to alcoholic neurodegeneration that was previously thought to primarily involve glutamatergic excitotoxicity mechanisms. Additional studies are needed to determine the contribution of DR3 and Fas to other mental and neurodegenerative diseases known to have increases in neuroimmune gene expression.

DR3 and Fas are known to increase proinflammatory cytokines as well as death receptor cascades in immune and cancer cells. In brain, DR3 and Fas are expressed primarily on neurons. Studies find Japanese encephalitis-induced neuronal apoptosis is dependent upon TRADD [33]. Among the TNF receptor family, DR3 and Fas signaling through TRADD and FADD to caspases is linked to neuronal apoptosis as well as axon degeneration-pruning [44]. In the rodent AIE model, we find increased active caspase 3 is correlated with loss of adult hippocampal neurogenesis [9]. Expression of active caspase 3 is colocalized with NeuN+ neurons in the adult hippocampal DG, consistent with caspase signaling contributing to neurodegeneration. In contrast, microglia [45] and astrocytes [46] are resistant to DR apoptosis and respond with proinflammatory gene induction [46]. Further, in our previous studies we found AIE increased adult microglial CD11b+IR [47], consistent with other studies finding ethanol exposure causes a persistent increase in microglial CD11B+IR [48, 49] in hippocampal DG, suggesting microglia do not degenerate. Thus, DR3-induced cell death is primarily neuronal. Knockdown of caspase-8 blocks Fas- and DR3-induced cell death, but not activation of kinases and NFκB signaling [50], further distinguishing DISC and proinflammatory brain responses. Our findings support induction of the entire caspase cascade. In the postmortem human Alzheimer’s disease hippocampus, there are increases in TRADD expression with the largest increases in patients with the highest number of neuritic plaques [51], whereas cortical FADD was reported to be lower in subjects with dementia and increased amyloid β pathology [52]. Previous studies have reported proinflammatory cytokines increased by alcohol exposure and in human postmortem AUD [3, 11]. However, these changes in gene expression were poorly linked to pathological mechanisms. In this study, we provide evidence of neuroimmune signaling persistently increasing DR signaling in AUD hippocampus through TL1A-DR3 and FasL–Fas, FADD and TRADD, and caspases-3, -7, -8, and -9 that likely contribute to apoptotic and other forms of AUD neurodegeneration that may be shared by other brain diseases associated with increased neuroimmune gene expression.

## Supplementary information

Supplemental Tables 1, 2, and 3

Supplemental Figure 1

Supplemental Figure 1 caption
