# Supplementary material for: Hippocampal TNF-death receptors, caspase cell death cascades, and IL-8 in alcohol use disorder
Source: Mol Psychiatry. 2020 Mar 5;26(6):2254–62. doi: 10.1038/s41380-020-0698-4 (PMC7483234; doi:10.1038/s41380-020-0698-4)
Supplement: Supplementary file 1 — Supplemental Tables 1, 2, and 3 [file 41380_2020_698_MOESM1_ESM.docx]

| **Supplemental Table 1.** Case characteristics of subjects used for immunohistochemical and RT-PCR analysis in the hippocampus of post-mortem human brain | | | | | | | |
| --- | --- | --- | --- | --- | --- | --- | --- |
| Subject ID | Classification | Age of Death | RIN | Clinical Cause of Death | Age of Drinking Onset | Lifetime Alcohol Consumption (kg) | Years Drinking |
| 301 | Control | 53 | 7.9 | Cardiac | 25 | 102 | 28 |
| 329 | Control | 48 | 6.9 | Cardiac | 25 | 17 | 23 |
| 335 | Control | 44 | 7.1 | Cardiac | 25 | 28 | 19 |
| 349 | Control | 43 | *Unknown* | Aspiration pneumonia | 25 | 13 | 18 |
| 395 | Control | 60 | 8 | Cardiac | 25 | *Unknown* | 35 |
| 453 | Control | 46 | 4.4 | Cardiac | 25 | 114.98 | 21 |
| 498 | Control | 24 | 6.2 | Cardiac | 20 | 14.60 | 4 |
| 582 | Control | 50 | 7.5 | Cardiac | 25 | 5.50 | *Unknown* |
| 629 | Control | 62 | 8.8 | Cardiac | 25 | 5.11 | 7 |
| 635 | Control | 50 | 8.6 | Cardiac | 25 | 18.25 | 25 |
| 210 | AUD | 51 | *Unknown* | GI haemorrhage | 17 | 1863.00 | 34 |
| 286 | AUD | 25 | 6.9 | Toxicity | 16 | 551.88 | 9 |
| 287 | AUD | 50 | 7 | Cardiac | 18 | 2452.80 | 32 |
| 297 | AUD | 44 | 7.9 | Cardiac | 20 | 638.75 | 10 |
| 351 | AUD | 42 | 8 | Toxicity | 18 | 1471.68 | 24 |
| 591 | AUD | 45 | 7.9 | Respiratory | 15 | 1799.45 | 29 |
| 643 | AUD | 61 | 6.1 | Cardiac | 16 | 8051.54 | 43 |
| 654 | AUD | 49 | 6.4 | Cardiac | 16 | 1011.78 | 33 |
| 670 | AUD | 49 | 6.2 | Cardiac | 14 | 613.20 | 35 |
| 679 | AUD | 61 | 8.3 | Cardiac | 17 | 5621.00 | 44 |
| RIN: RNA integrity number. Mean age of drinking onset for moderate drinking controls was 25 (±1) years and for individuals with alcohol use disorder (AUD) was 17 (±1) years. AUD individuals tended to demonstrate an earlier age of drinking onset in comparison to moderate drinking controls. . Humans with AUD typically start drinking alcohol at an early age and ANOVA analysis indicated significant differences in “age began drinking” (F_1,20_ = 139.65, *p* = 0.000) and in “lifetime alcohol” consumption (F_1,19_ = 13.60, *p* = 0.004) between controls and AUD. All subjects were males. | | | | | | | |

| **Supplemental Table 2.** List of primary antibodies used for immunohistochemistry in the hippocampus of rat and post-mortem human brain | | | | | |
| --- | --- | --- | --- | --- | --- |
| Antibody | Isotype | Source/ Purification | Dilution | Company, Catalog Number | Validation |
| TNFRSF25/DR3 | Rabbit IgG | Polyclonal | 1:100 | Lifespan Biosciences, Inc., LS-B7731 | WB, IHC (Lifespan Biosciences) |
| TNFSF15/TL1A | Rabbit IgG | Polyclonal | 1:120 | Bioss Inc., bs-5092R | WB, IHC (Biosis) |
| FADD | Rabbit IgG | Polyclonal | 1:50 | Abcam Inc. ab24533 | WB, IHC^1^ |
| pFADD (phospho Ser194) | Rabbit IgG | Polyclonal | 1:50 | Santa Cruz Biotechnology, Inc., sc-12439 | WB preabsorption^2^ |
| TRADD | Rabbit IgG | Polyclonal | 1:50 | Novus Biologicals, NB100-56169 | WB, IHC^3^ |
| Active (Cleaved) caspase 3 | Rabbit IgG | Monoclonal | 1:1200 | Cell Signaling Technology, #9661 | WB, siRNA^3^ |
| Active (Cleaved) caspase 7 | Rabbit IgG | Polyclonal | 1:50 | Biorbyt, orb159339 | WB (Biorbyt) |
| Active (Cleaved) caspase 8 | Rabbit IgG | Polyclonal | 1:400 | Novus Biologicals, NB100-56116 | WB, siRNA^3^ |
| Active (Cleaved) caspase 9 | Rabbit IgG | Polyclonal | 1:50 | Biorbyt, orb159342 | WB, IHC (Biorbyt) |
| Fas | Rabbit IgG | Polyclonal | 1:100 | Abcam Inc. ab82419 | IHC-KO^4^ |
| FasL | Rabbit IgG | Polyclonal | 1:100 | Abcam Inc. ab15285 | WB, IHC^5^ |
| NF-κB p65 (phospho S536) | Rabbit IgG | Polyclonal | 1:100 | Abcam Inc. ab86299 | WB, IHC^6^ |
| IL-8 | Mouse IgG | Monoclonal | 1:500 | Abcam Inc. ab18672 | WB, IHC^7^ |
| Validation information includes references as well as methods used for validation and source. ^1^Jaiswal, M.K., Agrawal, V., Maller, T., Gilman-Sachs, A. et al. (2013). Regulation of apoptosis and innate immune stimuli in inflammation-induced preterm labor. J. Immunology, 191, 5702-5713. ^2^Ramos-Miguel, A., Garcia-Fuster, M.J., Callado, L.F., La Harpe, R.et al. (2009). Phosphorylation of FADD (Fas-associated death domain protein) at serine 194 is increased in the prefrontal cortex of opiate abusers: Relation to mitogen activated protein kinase, phosphoprotein enriched in astrocytes of 15 kDa, and Akt signaling pathways involved in neuroplasticity. Neuroscience, 161, 23-38. ^3^Chang, X., Wang, L., Wang, Z., Wu, S. et al. (2017). TRADD mediates the tumor necrosis factor-induced apoptosis of L929 cells in the absence of RIP3. Sci. Rep, 7. ^4^Teodorczyk, M., Kleber, S., Wollny, D., Sefrin, J. P. et al. (2015). CD95 promotes metastatic spread via Sck in pancreatic ductal adenocarcinoma. Cell Death Differ, 22, 1192-1202. ^5^Liu, G., Yuan, Y., Long, M., Luo, T. et al. (2017). Beclin-1-mediated Autophagy Protects Against Cadmium-activated Apoptosis via the Fas/FasL Pathway in Primary Rat Proximal Tubular Cell Culture. Sci Rep, 7. ^6^Xiao, F., Zheng, R., Yang, D., Cao, K. et al. (2017). Sex-dependent aortic valve pathology in patients with rheumatic heart disease. PLoS One, 12, e0180230. ^7^Levina, V., Su, Y., Nolen, B., Liu, X. et al. (2008). Chemotherapeutic drugs and human tumor cells cytokine network. Int. J., Cancer, 123, 2031-2040. | | | | | |

| **Supplemental Table 3.** Primers used for mRNA quantification by RT-PCR in the hippocampus of post-mortem human brain | | |
| --- | --- | --- |
| *Gene* | Forward | Reverse |
| *FasL* | CTT TCC TCC TTG ATT TCT TCA TTC A | GCT TAC ATA GGC AGT ACG TGG A |
| *FasR* | GTC TCC TGC GAT GTT TGG C | TTC AAG GAA AGC TGA TAC CTA TTT C |
| *DR3* | GAG TTG AGA CGC AGG AGG CTA | GTT GGG GGA GGG ACA CTG AT |
| *TL1A* | CAA AAT GGC AAC TGG CTT TCA TA | CCT CCC TTT CCC TCA TCT GAC |
| *FADD* | GAT TGG AGA AGG CTG GCT CG | ATC AGG ACG CTT CGG AGG TA |
| *TRADD* | TTG CTG AAC CCC TGT CCA TC | CCT GGG GAA GGC AAT CAA CT |
| *IL-8* | ATA AAA AGC CAC CGG AGC ACT | ACA GTG AGA TGG TTC CTT CCG |
| *Caspase 3* | CCC AGG CCG TGA GGA GTT A | TTA ATG AGA ATG GGG GAA GAG GC |
| *Caspase 7* | TTT GTA GAG CGA GGG GCC AA | TGG AAG AGC CCA AAG CGA C |
| *Caspase 8* | AGG CAC AGA GAT TAA GTC CAT T | GAC ACA CAG ACT CGA ATG CC |
| *Caspase 9* | GGC CCT GGA TGT TAG GAT GGA T | GAC TTG GAA AGG TCA CAG AAG G |
| *NF-κB p65* | CAT TGG TGG TAG AGA GCT GGG | CGC TGG CTA GTC CCT CTT TG |
| β *actin* | GAT GCA GAA GGA GAT CAC TGC | ATA CTC CTG CTT GCT GAT CCA |
