# Supplementary figures and images for: Hippocampal TNF-death receptors, caspase cell death cascades, and IL-8 in alcohol use disorder
Source: Mol Psychiatry. 2020 Mar 5;26(6):2254–62. doi: 10.1038/s41380-020-0698-4 (PMC7483234; doi:10.1038/s41380-020-0698-4)

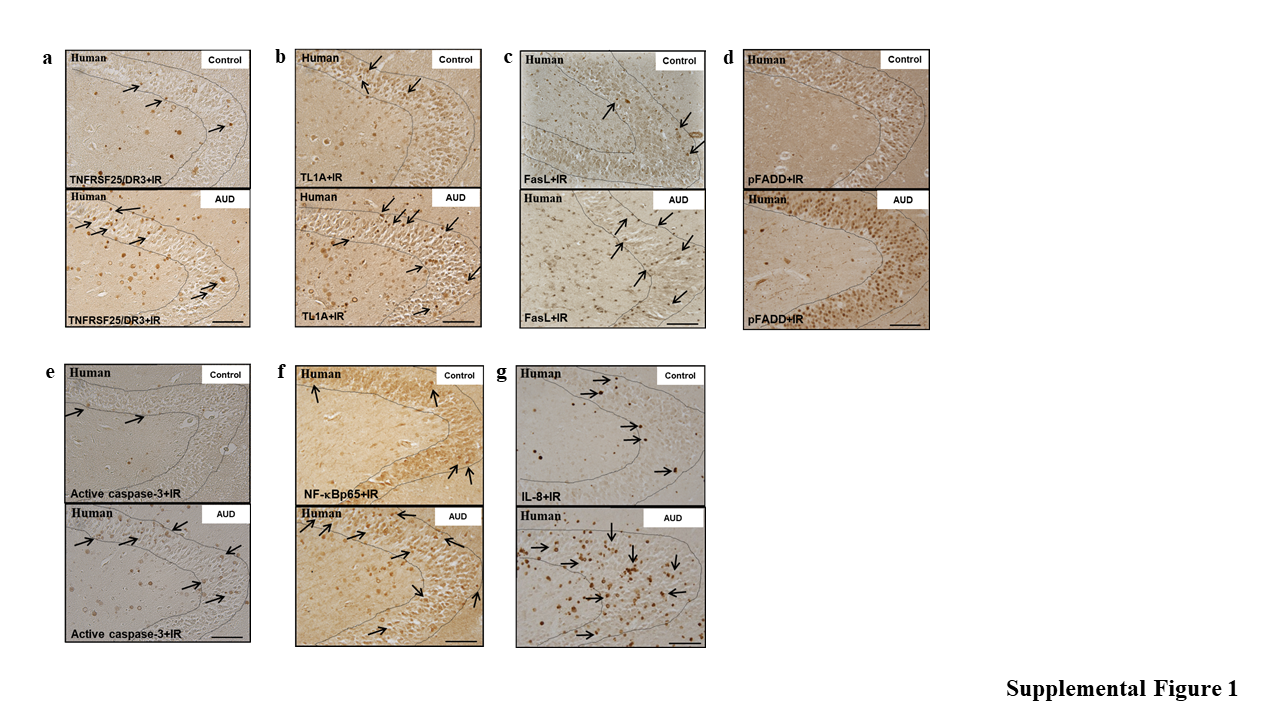

Supplement: Supplementary file 2 — Supplemental Figure 1 [file 41380_2020_698_MOESM2_ESM.tif]
