# Supplementary material for: Hippocampal TNF-death receptors, caspase cell death cascades, and IL-8 in alcohol use disorder
Source: Mol Psychiatry. 2020 Mar 5;26(6):2254–62. doi: 10.1038/s41380-020-0698-4 (PMC7483234; doi:10.1038/s41380-020-0698-4)
Supplement: Supplementary file 3 — Supplemental Figure 1 caption [file 41380_2020_698_MOESM3_ESM.docx]

**Supplemental Figure 1. Representative immunohistochemical staining in the hippocampus of post-mortem human control and alcohol use disorder (AUD) subjects.** Photomicrographs of (a) TNFRSF25/DR3, (b) TL1A, (c) FasL, (d) pFADD, (e) active caspase-3, (f) phosphorylated NF-κB p65, and (g) IL-8 immunohistochemistry in control and AUD subjects. Bar scale = 50 μm.
